# Epidemiology of intussusception in sub-Saharan Africa

**DOI:** 10.11604/pamj.supp.2021.39.1.30287

**Published:** 2021-07-27

**Authors:** Jason M Mwenda, Jacqueline E Tate, Umesh D Parashar

**Affiliations:** 1 World Health Organization (WHO) Regional Office for Africa, Brazzaville, Republic of Congo,; 2 Centers for Disease Control and Prevention, Atlanta, Georgia, USA

**Keywords:** intussusception, rotavirus, vaccine

## Abstract

This supplement contains the findings from intussusception surveillance conducted in 9 countries. These articles provide information on the age distribution of intussusception in the first year of life with cases peaking at 4-6 months of age, highlight the high proportion of cases in most, but not all, countries that undergo surgery and often require bowel resection for the treatment of intussusception, and show the variability of treatment outcomes in different countries. These data will be important for improving diagnosis and treatment of intussusception in young children in sub-Saharan Africa.

## Editorial

Intussusception, a condition in which one section of the bowel invaginates into a distal section, is the most common type of bowel obstruction in infants and young children globally [1]. In the late 1990s, a live oral rotavirus vaccine (RotaShield, Wyeth Lederle Vaccines) was associated with intussusception [2-4]. This vaccine was only introduced in the United States and was subsequently withdrawn from use approximately one year after its introduction due to this association with intussusception [5]. At the time, sparse data were available on intussusception in young children globally including on the age distribution of cases in the first year of life when rotavirus vaccine was to be administered. In the subsequent years, several studies were conducted to describe the epidemiology of intussusception in infants and young children [6-8]. These studies showed that the incidence of intussusception naturally increased sharply from 2 to 6 months of age at the same time that doses of rotavirus vaccine were administered. Reanalysis of the data on intussusception following rotavirus vaccination in the United States found that the apparent higher risk of intussusception among children vaccinated at older compared to younger ages may be attributed, at least in part, to this underlying changing age distribution during the vaccination window [9].

In the early 2000s, two new rotavirus vaccines (Rotarix, GSK Biologicals and RotaTeq, Merck and Co.) were developed for the global market and large clinical trials to assess the risk of intussusception were conducted largely in high and middle income countries [10, 11]. These trials found no increased risk of intussusception which led to the introduction of these vaccines in many countries. However, post-licensure evaluations were recommended to assess lower levels of risk of intussusception associated with these vaccines and to provide data from regions where safety trials were not conducted [12]. A significant association of approximately 1 to 6 additional cases of intussusception per 100,000 infant´s vaccinated, but substantially smaller in magnitude than with the previous rotavirus vaccine, was observed between these newly available rotavirus vaccines and intussusception in some high- and middle-income countries [13-20]. However, given that the benefits of vaccination in terms of prevented rotavirus hospitalizations and deaths compared to the small increased risk of intussusception, global and national policy makers have continued to support the use of rotavirus vaccines due to these benefits [21].

More recent data showed no increased risk of intussusception following rotavirus vaccination in a pooled analysis of data from seven countries in sub-Saharan Africa and in a similar analysis across eight hospitals in South Africa [22, 23]. Reasons for the absence of risk in low-income countries are not known but may be related to a lower age at vaccination, less replication of the vaccine virus in the child´s gut due to lower immunogenicity and co-administration of oral polio vaccine, or differences in infant microbiome, nutrition, weaning practices, or maternal antibody levels. While the finding of no increased risk of intussusception was reassuring, especially given the high burden of rotavirus disease across the continent, the planning and execution of these post-licensure evaluations in many countries highlighted the limited data available on intussusception in sub-Saharan Africa. In 2018, two Indian-manufactured rotavirus vaccines (Rotavac, Bharat Biotech and Rotasiil, Serum Institute) were pre-qualified by WHO [24]. Several post-licensure evaluations of Rotavac in India found no increased risk of intussusception associated with this vaccine [25-27]. In Africa, post-licensure evaluations of both vaccines are currently ongoing in early adopting countries of these vaccines.

A previous review of intussusception in children in sub-Saharan Africa identified 16 studies in 7 countries over a 34-year period from 1980 to 2014 with only 6 studies with data from the time period when rotavirus vaccines were available on the continent [28]. Methodologies used in these studies varied, as did the populations under surveillance, case identification methods, and data elements collected. To help fill in the gaps in the understanding of the epidemiology of intussusception in sub-Saharan Africa, intussusception surveillance has continued in Africa. This supplement contains the findings from intussusception surveillance conducted in 9 countries. These articles provide information on the age distribution of intussusception in the first year of life with cases peaking at 4-6 months of age, highlight the high proportion of cases in most, but not all, countries that undergo surgery and often require bowel resection for the treatment of intussusception, and show the variability of treatment outcomes in different countries. These data will be important for improving diagnosis and treatment of intussusception in young children in sub-Saharan Africa. Furthermore, two new Indian-manufactured rotavirus vaccines have recently been pre-qualified and recommended for use by the World Health Organization and there is a need to monitor these vaccines for an association with intussusception as they are introduced in Africa [24]. The information in these articles will also help to plan post-licensure evaluations for these newly available rotavirus vaccines.

**Disclaimer:** the findings and conclusions in this report are those of the authors and do not necessarily represent the official position of the Centers for Disease Control and Prevention (CDC) or the World Health Organization.
